# The epidemiology and treatment of anal fissures in a population-based cohort

**DOI:** 10.1186/1471-230X-14-129

**Published:** 2014-07-16

**Authors:** Douglas W Mapel, Michael Schum, Ann Von Worley

**Affiliations:** 1 Lovelace Clinic Foundation, 2309 Renard Place SE, Albuquerque NM 87106, New Mexico, USA

**Keywords:** Anal fissure, Epidemiology, Risk factors, Comorbidity, Treatment

## Abstract

**Background:**

Anal fissure (AF) is regarded as a common problem, but there are no published epidemiologic data, nor information on current treatment. The purpose of this study was to examine the incidence, associated comorbidities, and treatment of AF in a population-based cohort.

**Methods:**

We conducted a retrospective analysis of all persons who were enrolled in one large regional managed care system and treated for AF during calendar years 2005–2011. All persons aged 6 years or older who had a clinic, hospitalization, or surgical procedure associated with AF were identified from utilization data. To identify comorbidities associated with AF, each case was matched by age and gender to 3 controls.

**Results:**

There were 1,243 AF cases, including 721 (58%) females and 522 (42%) males; 150 (12%) of the cases occurred in children aged 6–17 years. The overall annual incidence was 0.11% (1.1 cases per 1000 person-years), but ranged widely by age [0.05% in patients 6–17 years to 0.18% in patients 25–34 years]. The incidence also varied by sex, and was significantly higher among females 12–24 years, and among males 55–64 years (*P* < 0.001). Comorbidities associated with AF included chronic constipation (prevalence 14.2% vs 3.6%), hypothyroidism (14.7% vs 10.4%), obesity (13.0% vs 7.7%), and solid tumors without metastasis (5.2% vs 3.7%) (*P* < 0.001 for all comparisons). A total of 448 were dispensed a topical prescription medication, 31 had botulinum toxin injection, and only 13 had lateral internal sphincterotomy.

**Conclusions:**

AF is a common clinical problem, and the incidence varies substantially by age and sex. Constipation, obesity, and hypothyroidism are associated comorbidities. Most patients are prescribed topical treatments, although it appears that many prescriptions are never filled. Surgical interventions for AF including botulinum toxin and lateral internal sphincterotomy are uncommon.

## Background

An anal fissure (AF) is a small break or tear in the skin of the anal canal, which typically runs from below the dentate line to the anal verge, and is usually situated in the posterior midline [1,2]. AF causes severe pain and bleeding with bowel movements, and is associated with spasm of the internal anal sphincter which may lead to reduction of blood flow and delayed healing [2]. Most AF are minor and thought to heal spontaneously, but those that are still symptomatic after 4 to 6 weeks are often referred to as chronic AF [1,2].

General surgeons and colon & rectal surgery specialists regard AF as a common problem in adults and children [3,4], but data on the epidemiology of this disease are very rare [5]. In a search of the PubMed database using the key words ‘anal fissure’ and ‘epidemiology’, ‘incidence’, or ‘prevalence’, we could not find any articles published within the last 30 years with population-based data on the incidence of AF.

Although the pathogenesis of AF is still uncertain, it is thought that most fissures are initiated by direct trauma from passage of hard stools or diarrhea [1-3]. However, in one review of the etiology of AF only 25% of patients with AF had chronic constipation [6]. In a prospective study of 165 women in the last three months of pregnancy and first 10 weeks post-partum, only 2 had AF before delivery, but 25 (15%) had AF afterwards, with half occurring more than 2 weeks after the delivery [7]. The finding of multiple fissures or AF in an unusual lateral position can be associated with Crohn’s disease, ulcerative colitis, HIV infection, neoplasia, syphilis, and tuberculosis [8].

Lateral internal sphincterotomy (LIS), accomplished by a radial incision in the anoderm followed by division of the internal sphincter muscles, has long been regarded as the surgical procedure of choice for AF [1]. Surgical interventions have had consistently higher success rates (approximately 89%) than any form of medical therapy, but are expensive and have a substantial risk of long-term anal incontinence [9,10]. Over the last three decades several topical or injected medications have been proposed as pharmacological or ‘chemical sphincterotomy’ alternatives to surgery [1,2,9]. Topical agents that relax the internal anal sphincter and increase blood flow to the affected area, such as nitroglycerin ointments or calcium channel blockers including diltiazem or nifedipine, have been proven in randomized placebo-controlled trials to be effective in reducing healing time and success rates [11-18]. Other treatments such as botulinim toxin injections that directly block anal sphincter hypertonia are marginally better than placebo in meta-analyses of current randomized trials, but success appears to be highly variable [9]. Most recent reviews on AF management, plus the current guidelines of several international societies of colon and rectal surgeons, acknowledge that while LIS is the ‘gold standard’ for AF treatment in terms of treatment success, it is reasonable to offer most patients medical treatments as first-line therapy [19]. How often these recommendations are followed among persons treated in the general population is not currently described in the medical literature.

The purpose of this study was to describe the epidemiology, risk factors, associated comorbidities, and current treatment of AF in a population-based cohort. To accomplish this, we performed a retrospective analysis of all persons aged 6 years and older who were diagnosed with AF and who were enrolled in a large integrated medical system in the Southwestern United States.

## Methods

The patients included in this retrospective historical cohort analysis were enrolled in one regional healthcare system based in Albuquerque, New Mexico, USA. Utilization data from this system are routinely de-identified and collected into an electronic database maintained by the Lovelace Clinic Foundation, a non-profit organization that specializes in health services research. All healthcare utilization and outpatient pharmacy fills for all members are captured by this database, regardless of where the utilization occurs. The system had an average annual enrollment of over 220,000 during the 7-year study period (January 1, 2005 and December 31, 2011). This project was approved and monitored by an independent human research review board (Ethical and Independent Review Services® 12072-01c).

For study inclusion, patients were required to have one or more visits or procedures during the study period associated with an ICD-9 diagnosis code of 565.0 (anal fissure). The date of the first visit associated with a 565.0 diagnostic code is referred to as the date of first diagnosis; these patients are referred to as incident AF cases. Patients may be an incident AF case only one time, and all of their subsequent visits are considered evidence of either continuation of the initial lesion or recurrence of AF. Persons who had follow-up visits for AF 12 weeks or more after the initial visit were defined as having chronic or recurrent AF. All cases had to be 6 years of age or older at the time of diagnosis for inclusion in the study.

The incidence of AF during the study period was calculated by comparing the number of incident AF cases diagnosed to the total person-years at risk within each age, sex, or ethnicity category. Patients could enroll or disenroll from the health plan in the middle of a calendar year, or otherwise not have a full 12 months of time at risk of developing AF, which creates a risk of bias that might be introduced by variable enrollment periods. Therefore, to reduce this bias, the incidence in person-years was calculated by comparing the number of AF cases occurring during a calendar year by the total person-months of enrollment during that period divided by 12.

Case–control matching was used to help identify comorbidities associated with anal fissures. Each anal fissure case was matched to 3 similar patients who did not have anal fissure. The matching criteria included:

• age at the time of diagnosis/index date [±two years]

• sex

• encounter type [ambulatory (clinic) visit, Emergency Department, or inpatient]

• diagnosis month [+/- 6 months]

• enrolled months after index date [+/- 2 months]

The Elixhauser system for capturing prognostically significant comorbidities was used for both the case and control groups [20]. To be included in the case–control analysis, patients had to have 12 months of continuous enrollment data available just prior to the first AF diagnosis date.

The prescription drugs are identified in the database by National Drug Code (NDC) identifiers, and all prescription drugs dispensed within one week of the initial diagnosis were captured for each AF patient. Procedures associated with AF were identified using Current Procedural Terminology (CPT) codes.

One limitation of the database is that it does not capture prescription medications that have to be formulated by compounding pharmacists because compounded medications do not have specific NDC codes and because these products are not a part of the insurance benefit covered by the health plan. The commercial 0.4% product (Rectiv®) was not available during the study period. However, discussions with local pharmacists confirmed that some AF patients during the study period were prescribed the 2% nitroglycerin ointment with instructions to either provide it to the compounding pharmacist to dilute to 0.2% or 0.4%, or for the patient to mix it with topical lidocaine or other products prior to application. Therefore, we assumed that all prescriptions for the topical 2% nitroglycerin product dispensed at the time of an AF visit were intended for the treatment of AF. All drugs that were dispensed to cases within one week of the index date, and all procedures associated with AF diagnoses, were captured and cross-referenced to find any patients with AF who may have been missed by the initial search.

To examine the frequency of initial treatment with compounded agents, and to capture the physician recommendations for non-prescription drug treatments, we conducted a medical record review and abstraction. A majority of the patients during the study period received their primary and specialty care through physicians affiliated with ABQ Health Partners® (ABQHP), which in 2008 began implementing an electronic medical record (EMR). For the medical record abstraction, all AF cases included in the ABQHP’s EMR system in the last two years of the study period were identified, and any information related to treatment for AF reviewed. A data abstraction instrument and protocol were developed by an experienced professional medical record abstractor, and the specific items to be abstracted verified by our surgical consultants.

### Statistical methods

All categorical variables were compared using Chi-square statistics with the Yates correction. Normally distributed means were compared using the Student’s t-test, and non-parametric continuous data were compared using the Wilcoxon rank-sum test. McNemar’s test was used for the case–control comparisons. All data processing and analyses were conducted using SAS® software, and the medical record abstraction data were compiled using Microsoft Access®.

## Results

**Figure 1:**
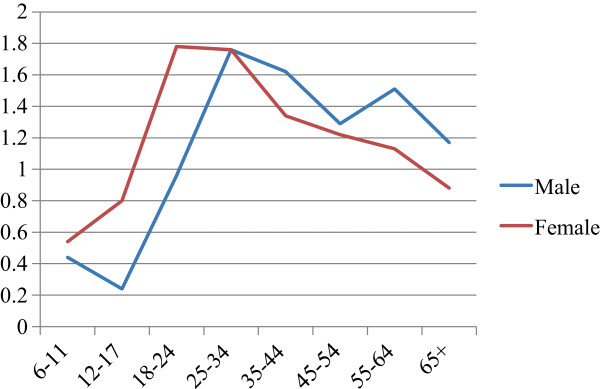
Incidence of anal fissure (cases per 1000 persons per year) by sex and age group.

A total of 1243 persons met all study inclusion criteria (Table 1). More women (N = 721, 58% of cohort) were diagnosed with AF than men. This health system enrolls a higher proportion of women overall (55.8% during the study period), and after calculation of the incidence of AF by person-years at risk, the overall difference in AF by sex did not reach statistical significance (*p* = 0.08) (Table 2). However, there were several important differences between men and women by age strata (Figure 1 and Table 2). Women from 12–24 had significantly higher incidence of AF than males at those age, while men age 55–64 had significantly higher AF incidence than women. Accordingly, the mean age among women with AF was 40.9 years and among men was 46.6 years (*p* < 0.001).

**Table 1:** Demographic characteristics of the anal fissure study cohort

	**Male**		**Female**	
**Description**	**N**	**%**	**N**	**%**
**Sex** (row%)	522	42.0%	721	58.0%
**Mean age** (years)*	46.6		40.9	
**Age categories** (column%)				
Age 6 - 11	34	6.5%	48	6.7%
Age 12 - 17	20	3.8%	48	6.7%
Age 18 - 24	38	7.3%	107	14.8%
Age 25 - 34	63	12.1%	134	18.6%
Age 35 - 44	81	15.5%	86	11.9%
Age 45 - 54	83	15.9%	101	14.0%
Age 55 - 64	94	18.0%	82	11.4%
Age 65+	109	20.9%	115	16.0%
**Ethnicity**				
Hispanic (row%)	249	42.9%	331	57.1%
Mean age (years)	44.6		38.1	
Non-Hispanic (row%)	273	41.2%	390	58.8%
Mean age (years)	48.3		43.3	

*Mean difference significant P < 0.001.

**Table 2:** Incidence of anal fissure (cases/1000 person-years) stratified by age group and sex

**Age group**	**Pooled incidence**	**P**^**†**^	**Male incidence**	**Female incidence**	**P**^**††**^
**6-11**	0.49	***	0.44	0.54	
**12-17**	0.52	***	0.24	0.80	***
**18-24**	1.47		0.96	1.78	***
**25-34**	1.76		1.76	1.76	
**35-44**	1.46		1.62	1.34	
**45-54**	1.25	**	1.29	1.22	
**55-64**	1.31	*	1.51	1.13	***
**65+**	1.01	***	1.17	0.88	
**Total**	1.10		1.04	1.14	

^**†**^Difference between 25–34 group and starred age groups significant at: *P < 0.01; **P < 0.001; ***P < 0.0001.

^**††**^Difference between sexes within this age group significant at: ***P < 0.0001.

Persons of Hispanic heritage comprised 46.6% of the overall cohort, which is consistent with the demographics of the regional population and this health system (Table 1). Overall, there was no difference between Hispanic and Non-Hispanic persons in the incidence of AF (1.09 and 1.10 cases per 1000 person-years, respectively). However, there was a higher incidence of AF among Hispanic persons in the 55–64 age group (1.69 versus 1.08 cases per 1000 person-years; *p* < 0.01), mostly due to a slightly higher occurrence among men (2.00 cases per 1000 person-years among Hispanic men age 55–64 years).

The case–control comparison of comorbid conditions identified several factors associated with AF (Table 3). As expected, the largest proportional difference was in diagnosed chronic constipation, which was more than 3 times higher in cases (14.0% versus 3.6%, *p* <0.001). Hypothyroidism (14.7% versus 10.4%, *p* <0.001) and obesity were also common and substantially increased among cases. Solid tumors without metastasis and weight loss were the only other conditions that reached statistical significance. Pregnancy was negatively associated with AF, which is the opposite of what was anticipated, but likely due to the preference among obstetricians in this system to use ICD-9 diagnosis code 664.6 (anal sphincter tear complicating delivery) instead of ICD-9 565 (anal fissure). Note that conditions that might contraindicate use of topical vasodilators, such as pulmonary vascular disease, are uncommon.

**Table 3:** Comparison of chronic comorbidities among anal fissure cases versus age- and sex-matched controls (1 to 3 matching)

	**Cases**		**Controls**		
**Description**	**N**	**%**	**N**	**%**	**P-value**
No. of patients* (% of cohort)	1062		3186		
**Elixhauser comorbidities:**					
AIDS	5	0.5%	7	0.2%	0.182
Alcohol abuse	24	2.3%	97	3.0%	0.183
Chronic blood loss anemia	12	1.1%	38	1.2%	0.870
Coagulopathy	14	1.3%	43	1.4%	0.939
Congestive heart failure	27	2.5%	93	2.9%	0.521
**Constipation**	**149**	**14.0%**	**114**	**3.6%**	**<0.001**
Deficiency anemias	87	8.2%	227	7.1%	0.250
Depression	149	14.0%	387	12.2%	0.110
Diabetes (no chr complication)	147	13.8%	402	12.6%	0.303
Diabetes (w/chr complications)	30	2.8%	117	3.7%	0.191
Drug abuse	29	2.7%	112	3.5%	0.216
Fluid and electrolyte disorders	56	5.3%	168	5.3%	1.000
Hypertension	344	32.4%	959	30.1%	0.161
**Hypothyroidism**	**156**	**14.7%**	**330**	**10.4%**	**<0.001**
Liver disease	34	3.2%	84	2.6%	0.332
Lymphoma	8	0.8%	17	0.5%	0.418
Metastatic cancer	12	1.1%	23	0.7%	0.203
**Obesity**	**138**	**13.0%**	**246**	**7.7%**	**<0.001**
Other neurological disorders	48	4.5%	161	5.1%	0.486
Paralysis	7	0.7%	24	0.8%	0.755
Peripheral vascular disease	37	3.5%	109	3.4%	0.923
Pregnancy	72	6.8%	276	8.7%	0.053
Psychoses	99	9.3%	256	8.0%	0.189
Pulmonary circulation disease	23	2.2%	60	1.9%	0.565
Renal failure	29	2.7%	84	2.6%	0.869
Rheumatoid arth./ col. vasc. dz	37	3.5%	120	3.8%	0.673
**Solid tumor - no metastasis**	**55**	**5.2%**	**118**	**3.7%**	**0.035**
Valvular disease	30	2.8%	111	3.5%	0.299
**Weight loss**	**40**	**3.8%**	**54**	**1.7%**	**<0.001**
**Any Elixhauser diagnosis**	**741**	**69.8%**	**2091**	**65.6%**	**0.013**

*The case–control comparison is limited to adults age 18 and older because the Elixhauser comorbidity classification system is not validated in children.

Of the 1243 patients in the cohort, 1109 (89.2%) were diagnosed during an ambulatory visit, 23 (1.8%) during a hospitalization, and the remaining 111 (8.9%) were diagnosed during a procedure such as colonoscopy. Among the ambulatory AF cases, 120 persons were initially diagnosed during an emergency department visit. Approximately two-thirds of the ambulatory AF cases (746 patients; 67.3%) did not have a billed follow-up visit after the initial diagnosis. Of those who did have a follow-up visit, 182 patients (16.4% of ambulatory cases) had only one follow-up visit, 73 (6.6%) had two follow-up visits, and the remaining 108 (9.7%) required three or more follow-up visits. Among the entire study cohort, 177 AF patients (14.2%) had a follow-up visit 12 weeks or more after the initial visit, indicating that they had chronic or recurrent AF.

At the time of the initial diagnosis, pharmacy utilization records indicate that 436 patients had one or more prescriptions filled for either a topical corticosteroid, lidocaine gel, nitroglycerine 2% ointment, diltiazem, or any combination of the four. Corticosteroids were by far the most common prescriptions filled (300 patients), followed by lidocaine (190 patients), nitroglycerine 2% ointment (93 patients), and diltiazem (2 patients). Most (346) patients initially filled only one type of treatment, and among those getting multiple prescription fills, lidocaine and nitroglycerine was the most commonly prescribed combination (48 patients).

Only 3 patients had a procedure for AF prior to getting a medical prescription therapy: one had a botulinim toxin (BT) injection as their initial treatment, and two persons had LIS as their initial treatment. All of the others who were treated with BT injections and 4 of the patients who had LIS waited at least 12 weeks after their initial diagnosis before getting their procedure. Of the 31 persons who had BT injections, 11 required at least one subsequent additional BT treatment. Of the 13 patients who had a LIS, only one required a second LIS procedure.

Medical record abstraction was accomplished for 179 patients who were diagnosed in the last 2 years of the study period and who had any documentation of their AF evaluation available in the EMR. The majority (54.8%) were initially diagnosed by a surgeon, but 61 patients (34.1%) were diagnosed by a primary care physician, and 8 AF patients (4.5%) were diagnosed by a gastroenterologist. Recommended treatments and prescriptions that were documented in the records are listed in Table 4. A total of 115 patients (64.2%) were recommended at least one prescription medication at the time of the initial diagnosis, which is substantially higher than the percent of ambulatory patients documented to have a prescription fill (39.3%). Compounded 0.4% nitroglycerine, which cannot be identified as dispensed in the utilization database, was a common initial treatment (21.8%). No patient was prescribed a compounded ointment containing a calcium channel blocker at the initial visit, but five patients (4 nifedipine, 1 diltiazem) were prescribed one on a follow-up visit.

**Table 4:** Type of medication prescribed at time of initial diagnosis*

	**N (% of abstracted)**
Nitroglycerine 0.4% (compounded)	39 (21.8%)
Nitroglycerine 2% (topical ointment)	29 (16.2%)
Lidocaine (topical ointment or gel)	70 (39.1%)
Corticosteroid (topical - all forms)	37 (20.1%)
Cyclobenzaprine tablets	7 (3.9%)
Topical antibiotic	4 (2.2%)
Diphenhydramine cream	2 (1.1%)

*Results from 179 patients with medical record abstraction.

Documentation of recommendations for non-prescription (over-the-counter) medications was recorded in the medical records of 65 patients (36.3%) either on the initial visit or during follow-up (Table 5). The majority (119 patients; 66.5%) of those abstracted also had non-medication recommendations such as sitz baths, dietary modifications, and adequate fluid intake.

**Table 5:** Over-the-counter (OTC) medications and non-medication treatments recommended at any time during the study period*

	**N (% of abstracted)**
Stool softeners	30 (16.8%)
Laxatives	39 (21.8%)
OTC topical pain relievers (e.g., Preparation H®, Anusol®)	11 ( 6.1%)
Total patients with a documented OTC medication recommendation	65 (36.3%)
Specific diet recommendations (e.g., increased fiber in diet)	96 (53.6%)
Total with ≥1 non-pharmaceutical treatment recommendations	119 (66.5%)

*Results from 179 patients with medical record abstraction.

## Discussion

Most surgeons and published expert opinions describe AF as ‘common’, but systematically collected incidence data on AF are not available. Extrapolation of the incidence rates in this cohort to the 2010 US census population (292.7 million aged ≥6 years), with adjustment by age and sex, indicates that there are approximately 342,000 new AF cases diagnosed in the US each year. This is similar to the annual incidence of appendectomies in the US (approximately 280,000 cases per year, or 0.7 to 1.7 cases per 1000 person-years depending on age) and in Ontario, Canada (approximately 0.75 appendectomies per thousand person-years) [21-25]. The overall incidence of 1.1 per 1000 person-years translates to an average life time risk of 7.8%, and thus AF is indeed a common problem.

Our analysis reveals some important details about the variation in AF incidence by age and sex. Women had a higher overall incidence (1.14 cases per thousand person-years), than men (1.04 cases per thousand person-years), but the difference did not reach statistical significance. Among women the peak incidence occurred during adolescence and young adulthood, but among men the incidence was highest during middle age. We had anticipated that women of child-bearing age would have a higher incidence of AF based on previously published studies among pregnant women, but we found that in this health system pregnancy-related fissures were more likely to be diagnosed and coded as anal tears (ICD-9 diagnosis code 664.6). This is probably a reasonable distinction since most delivery related tears are anterior and a result of perineal injury from childbirth, while most AF are posterior and thought to be due to relative insufficiency in blood flow. Because this is a retrospective survey we can only speculate about the reasons for the fluctuations in incidence by age and gender. However, the case–control analysis confirms a strong association between constipation and AF, and between other conditions associated with constipation (e.g., hypothyroidism, obesity). The variation in AF incidence by age and sex also tends to follow changes in constipation incidence by age and sex [26].

There are important limitations to this study that should be considered. First, we relied on ICD-9 coding to identify AF cases, which introduces the potential for misclassification errors. We found in the chart review of 179 cases that a physician had documented a diagnosis AF in every case, so the ICD-9 code for AF (565.0) was specific, but we do not know the sensitivity of this case identification system because we are not able to go back and reexamine every potential AF patient. Other research suggests that the accuracy of AF diagnosis depends greatly on the skills of the examining physician. One study from Spain demonstrated that surgeons can identify AF with very high reliability, but primary care providers were much less likely to correctly identify an AF lesion [27].

Another limitation is that the population demographics in the study area are substantially different than those in other parts of the US. Although we did not find a substantial difference between Hispanics and Non-Hispanics, with the possible exception of middle aged men, it is possible that other ethnic or racial groups have a different risk for AF.

Our risk estimates for the comorbidities associated with AF are likely to be biased by the fact that patients with AF were very likely to be asked about constipation and other gastrointestinal problems, while the controls were less likely to be asked about constipation because they were being seen for any complaint other than AF. In fact, the prevalence of constipation in our control population was much lower than the estimates from population surveys [26].

Finally, it is very difficult to capture utilization of compounded prescription drugs that are not paid for by the insurance plan. Our medical record review confirmed that substantial numbers of AF cases (64.2%) were given prescriptions for compounded nitroglycerine and calcium channel blockers, but our review of the pharmacy utilization data indicates that actual prescription fills were much lower than this. For example, 16.2% of patients in the chart review were given a prescription for nitroglycerin 2% cream, but only 7.5% of patients in the overall cohort actually had a prescription for nitroglycerine 2% cream filled. We suspect that many of these prescriptions were never filled because compounded preparations are not available in all pharmacies, and because many patients find their high cost unaffordable and not covered by their insurance benefit.

## Conclusions

We have demonstrated that AF is a problem that many persons of all ages suffer from, and something that most surgeons and many primary care providers will be asked to diagnose and treat. The fluctuations in incidence and the associated risk factors suggest areas that could be further investigated for better understanding of the pathogenesis and prevention of AF. There are also some aspects of AF treatment that could also be improved. As new products for AF treatment become available, we anticipate that education and investigations into clinical management will increase, and hopefully more physicians will be able to effectively diagnose and manage AF patients.

## Abbreviations

ABQHP: ABQ Health Partners®; AF: Anal fissure; BT: Botulinum toxin; CPT: Current procedural terminology; EMR: Electronic medical record; HIV: Human immunodeficiency virus; ICD-9: The International Classification of Diseases, 9th Revision, Clinical Modification; LIS: Lateral internal sphincterotomy; NDC: National drug code; USA: United States of America.

## Competing interests

This project was funded by a grant from Ventrus Pharmaceuticals, Inc. to the Lovelace Clinic Foundation. Ventrus Pharmaceuticals is involved in the development of topical treatments for anal fissure. Employees of Ventrus Pharmaceuticals were consulted about the original study concept, but were not involved in data collection, analyses, or writing of this manuscript. None of the authors has a personal or financial conflict of interest in this manuscript.

## Authors’ contributions

Dr. DM designed the study, analyzed and interpreted the data, and drafted the manuscript. Dr. MS participated in the design of the study, collected, analyzed and interpreted the data, and edited the manuscript. Ms. AVW participated in the design of the study, collected, analyzed and interpreted the data, and edited the final manuscript. All authors read and approved the final version of the manuscript.

## Pre-publication history

The pre-publication history for this paper can be accessed here:

http://www.biomedcentral.com/1471-230X/14/129/prepub
